# Differential effects of cholesterol levels on cognition according to body mass index in Parkinson’s disease

**DOI:** 10.1186/s13195-023-01326-2

**Published:** 2024-01-31

**Authors:** Seong Ho Jeong, Seok Jong Chung, Han Soo Yoo, Jin Ho Jung, Jong Sam Baik, Young H. Sohn, Phil Hyu Lee

**Affiliations:** 1 Department of Neurology, Inje University Sanggye Paik Hospital, Seoul, South Korea; 2 Department of Neurology, Yonsei University College of Medicine, 50 Yonsei-Ro, Seodaemun-Gu, Seoul, 03722 South Korea; 3 Department of Neurology, Yongin Severance Hospital, Yonsei University Health System, Yongin, South Korea; 4 Department of Neurology, Gangnam Severance Hospital, Yonsei University College of Medicine, Seoul, South Korea; 5 Department of Neurology, Inje Universitiy Busan Paik Hospital, Seoul, South Korea; 6 Severance Biomedical Science Institute, Yonsei University College of Medicine, Seoul, South Korea

**Keywords:** Cholesterol, Body mass index, Parkinson’s disease, Cognition, Dementia

## Abstract

**Background:**

Cholesterol is an essential component of the neuronal cell membrane and is crucial for neuronal function; however, the role of cholesterol levels in Parkinson’s disease (PD) is debatable. This study investigated the complex relationship between total cholesterol (TC) levels, body mass index (BMI), and cognition in patients with PD.

**Methods:**

This study included 321 drug-naïve patients with PD who underwent dopamine transporter (DAT) imaging and baseline neuropsychological tests. Multivariate linear regression and Cox regression models were used to investigate the effect of TC levels on the composite score of each cognitive domain and dementia conversion after adjusting for covariates, respectively. Interaction analyses were performed to examine the interaction effect between TC levels and BMI on baseline cognition and dementia conversion.

**Results:**

TC levels and cognition showed no significant relationship after adjusting for potential confounders. A significant interaction effect between TC levels and BMI was observed in frontal/executive function and dementia conversion. Further analyses showed that TC levels were positively associated with frontal/executive function in the under-/normal weight group (*β* = 0.205, *p* = 0.013), whereas a negative relationship existed between TC levels and frontal/executive function in the obese group (*β* =  − 0.213, *p* = 0.017). Cox regression analyses also showed the differential effects of TC levels on dementia conversion according to BMI (under-/normal weight group: hazard ratio [HR] = 0.550, *p* = 0.013; obese group: HR = 2.085, *p* = 0.014).

**Conclusions:**

This study suggests a cross-over interaction between TC levels and BMI on cognitive symptoms in PD.

**Supplementary Information:**

The online version contains supplementary material available at 10.1186/s13195-023-01326-2.

## Background

Parkinson’s disease (PD) is a neurodegenerative disorder characterized by the loss of dopaminergic neurons in the substantia nigra, leading to motor symptoms such as tremors, rigidity, and bradykinesia [1]. Additionally, cognitive impairment, one of the most common non-motor symptoms in patients with PD, is debilitating and significantly affect patients’ and caregivers’ quality of life [2].

Cholesterol is a lipid molecule that is essential for the normal functioning of cell membranes and is involved in the production of several important signaling molecules in the brain [3, 4]. Ample evidence has suggested that cholesterol may play a role in the pathogenesis of PD and cognitive decline. Elevated cholesterol levels are associated with an increased risk of cognitive impairment and dementia, including Alzheimer’s disease (AD) and vascular dementia [5, 6], whereas the other studies showed contradictory results [7–9]. Similarly, the association between cholesterol levels and cognition in patients with PD has shown conflicting results in several studies. One study showed that elevated plasma low-density lipoprotein (LDL) cholesterol levels were associated with more preserved executive function in PD [10]. Another study showed no significant association between cholesterol levels and cognitive decline in advanced stages of PD [11].

The body mass index (BMI) is an important clinical factor that is related to presynaptic dopaminergic degeneration and cognitive impairment in patients with PD [12–14]. Moreover, BMI is closely associated with cholesterol levels [15, 16], and the effect of cholesterol levels on cognition differs according to BMI in the elderly [17, 18]. Despite the potential importance of cholesterol and BMI in cognitive function, little is known about how these factors interact in patients with PD. Understanding these relationships could help identify novel targets for therapeutic interventions to modulate cognitive function in patients with PD. Therefore, this study aimed to investigate the differential effects of cholesterol levels on baseline and longitudinal cognition according to BMI in patients with PD.

## Methods

### Participants

We reviewed the medical records of 321 consecutive drug-naïve patients with PD who had visited the outpatient clinic for movement disorders at the Severance Hospital between April 2009 and September 2015. PD was diagnosed according to the clinical diagnostic criteria of the United Kingdom PD Society Brain Bank. All patients exhibited nigrostriatal dopamine depletion on [^18^F] N-(3-fluoropropyl)-2β-carbonethoxy-3β-(4-iodophenyl) nortropane positron emission tomography (^18^F-FP-CIT PET), showing reduced dopamine transporter (DAT) availability in the posterior putamen compared to 26 healthy controls. All patients underwent brain magnetic resonance imaging (MRI) and a detailed baseline neuropsychological study. The severity of parkinsonian motor deficits was assessed using the Unified PD Rating Scale Part III (UPDRS-III) at the initial visit (drug-naïve status). Total cholesterol (TC) levels were measured in blood collected after a 10-h overnight fast. Patients with missing data on the baseline neuropsychological study and TC levels were excluded from the study. The baseline height and weight were measured in all patients at their initial visit and used to calculate the BMI. According to the revised Asia–Pacific BMI criteria of the World Health Organization for western Pacific regions [19], BMI was classified as follows for subgroup analyses: underweight, BMI < 18.5 kg/m^2^; normal weight, BMI ≥ 18.5 and < 23 kg/m^2^; overweight, BMI ≥ 23 kg/m^2^ and < 25 kg/m^2^; and obese, BMI ≥ 25 kg/m^2^. We divided the patients with PD into the following three groups: under-/normal weight, BMI < 23 kg/m^2^ (*n* = 138); overweight, BMI ≥ 23 and < 25 kg/m^2^ (*n* = 102), and obese, BMI ≥ 25 kg/m^2^ (*n* = 81). Underweight participants were pooled with the normal weight group because only 12 patients had a BMI < 18.5 kg/m^2^. All patients were investigated for their medical history and vascular risk factors, including hypertension and diabetes mellitus. We also investigated the types of statins used by the patients (atorvastatin, *n* = 58; simvastatin, *n* = 10; pitavastatin, *n* = 5; fluvastatin, *n* = 4; rosuvastatin, *n* = 22; and pravastatin, *n* = 4). Two movement disorder experts (Y.H.S. and P.H.L.) confirmed levodopa responsiveness and carefully assessed atypical parkinsonism during a mean period of 5.31 years. This study was approved by the institutional review board of Yonsei University College of Medicine (No. 4–2014–0637). Informed consent was waived due to the nature of retrospective study.

### Acquisition and quantitative analyses of ^18^F-FP-CIT PET

Image acquisition and quantitative analyses of ^18^F-FP-CIT PET data were performed according to the methods reported in our previous study (Supplementary Methods). Briefly, the striatum was divided into the anterior caudate, posterior caudate, anterior putamen, posterior putamen, ventral putamen, and ventral striatum. Dopamine transporter (DAT) availability of each striatal subregion was defined as (mean standardized uptake value of the striatal subregion volume of interests [VOIs] − mean standardized uptake value of the occipital VOI)/(mean standardized uptake value of the occipital VOI). Because DAT availability in the posterior putamen (DAT-PP) is most severely affected area in patients with PD, DAT activity in this region was included in the data analyses.

### Acquisition of fluid-attenuated inversion recovery (FLAIR) sequence images and grading of the white matter hyperintensities (WMHs)

Of the 321 enrolled patients, 244 (76.01%) underwent brain MRI at Severance Hospital using a 3.0-T scanner (Achieva; Philips Medical System, Best, Netherlands) with a 32-channel receiver array head coil at the initial assessment. Head motion was minimized with restraining foam pads provided by the manufacturer. The imaging protocol included FLAIR images (TR/TE, 9000–10,000/110–125 ms; section thickness, 5 mm; matrix, 256 × 256). The remaining 77 (23.99%) patients underwent brain MRI including FLAIR sequences at other hospitals before their referral to our hospital. Two neurologists (S.H.J. and S.J.C.) assessed the visual rating scale of WMHs using a semi-quantitative Scheltens scale [20]. The intra- and inter-rater reliabilities of the total WMHs were high (intraclass correlation coefficients = 0.984 and 0.966, respectively). If the score was discordant between the raters, the final score was determined by consensus.

### Neuropsychological evaluation

All participants underwent the Seoul Neuropsychological Screening Battery, a standardized neuropsychological battery that contains tests regarding attention/working memory, language, visuospatial function, memory, and frontal/executive function [21]. Standardized *z*-scores were available for all scorable tests based on age- and education-matched norms. We included the following tests: the digit span forward and backward and Stroop color reading test for the attention domain; the Korean version of the Boston Naming Test for the language domain; the copying item of the Rey-Osterrieth Complex Figure Test (RCFT) for the visuospatial domain; immediate recall, 20-min delayed recall, and recognition items of the RCFT and Seoul Verbal Learning Test for the memory domain; and the Controlled Oral Word Association Test for semantic (animal and supermarket) and phonemic fluency for the frontal/executive domain. A lower *z*-score in each test represents worse cognitive performance. A composite score was calculated for each cognitive domain by dividing the sum of *z*-scores by the number of tests. The Korean version of the Mini-Mental State Examination (MMSE) and Clinical Dementia Rating-Sum of Boxes (CDR-SOB) were used to assess the global cognitive performance.

### Assessment of dementia conversion

Of the 321 patients with PD, 15 were excluded due to changes in the status of statin use. Finally, 306 patients were included in the longitudinal analysis. The participants visited our outpatient clinic every 3–6 months, and movement disorder experts carefully assessed patients’ daily functioning, such as their ability to manage finances, use pieces of equipment, and cope in social situations, through a detailed history obtained from patients and caregivers [12]. In patients with complaints of cognitive decline or evidence of impairments in daily life due to cognitive changes, a detailed neuropsychological battery was subsequently conducted to diagnose Parkinson’s disease dementia (PDD) at level II in most patients. PDD was diagnosed using the clinical diagnostic criteria for probable PDD [22] based on the consensus between two neurologists and one neuropsychologist with evidence of abnormal activities of daily living (ADL), judged both clinically and based on instrumental ADL scales, whereas functional disabilities due to parkinsonism were not considered to be an impairment of complex ADL.

### Statistical analysis

To investigate the effect of TC levels per standard deviation (SD), we used individual z-transformed values of the TC levels in all analyses. To compare baseline demographic and clinical characteristics according to BMI, one-way analysis of variance with post hoc Bonferroni correction was used for continuous variables, and Pearson’s *χ*^2^ tests or Fisher’s exact tests were used for categorical variables. Multivariate linear regression analysis was used to determine the effect of the independent variables on the composite score of each cognitive domain after adjusting for age at symptom onset, sex, years of education, disease duration, hypertension, diabetes mellitus, statin use, DAT-PP, WMHs burden, BMI, and TC levels. We further tested whether there was an interaction effect between BMI and total cholesterol levels on the composite score of each cognitive domain after additional adjustment for the interaction term between BMI and total cholesterol levels. If the interaction term (BMI × TC levels) was significant, further subgroup analyses according to BMI (under-/normal weight, overweight, and obese groups) were performed. False discovery rate (FDR) was used to correct multiple tests for the five cognitive function domains.

Cognitive outcomes were assessed using the Kaplan–Meier survival analysis and Cox regression model for PDD conversion. The time from the onset of motor symptoms to the date of PDD conversion was assessed with Kaplan–Meier estimates in 306 patients with PD who were included in the final longitudinal analysis after categorizing the patients with PD into three groups according to cholesterol levels using a tertile-based approach (1st tertile group [TC levels < 172; *n* = 100], 2nd tertile group [172 ≤ TC levels < 197; *n* = 102], and 3rd tertile group [TC levels ≥ 197; *n* = 104]). The log-rank test was used to compare Kaplan–Meier plots among groups. To assess the effects of TC levels on PDD conversion, we estimated hazard ratios (HRs) and 95% confidence intervals (CIs) using Cox regression models while adjusting for age at symptom onset, sex, symptom duration, hypertension, diabetes mellitus, statin use, DAT-PP, WMHs burden, and baseline BMI. We further tested whether there was an interaction effect between BMI and total cholesterol levels on PDD conversion after additional adjustment for the interaction term between BMI and total cholesterol levels. The Bonferroni correction was applied for multiple comparisons of the five cognitive domains. If the interaction term (BMI × TC levels) was significant for the composite score of the cognitive domain, further subgroup analyses according to BMI (under-/normal weight, overweight, and obese groups) were performed.

Statistical analyses were performed using R software (v.4.0, http://www.r-project.org/). Results with *P* < 0.05 were considered statistically significant.

## Results

### Baseline demographic and clinical characteristics

Baseline demographic and clinical characteristics according to BMI group are summarized in Table 1. All clinical variables including age at symptom onset, proportion of women, UPDRS-III score, MMSE score, proportion of vascular risk factors including hypertension and diabetes mellitus, TC levels, proportion of statin use prior to PD diagnosis, WMHs burden score, and follow-up duration were comparable across the BMI groups. Years of education were longer in the overweight group than those of the obese group. The symptom duration prior to PD diagnosis was longer in the under-/normal weight group than that of the obese group. There were no differences between the BMI groups in the composite scores of any cognitive domain, including attention/working memory, language, visuospatial, memory, and executive functions, as well as CDR-SOB. Additionally, DAT availability in each striatal subregion was comparable among the BMI groups.

**Table 1:** Demographic characteristics in patients with Parkinson’s disease

	Under-/normal weight (*n* = 138)	Overweight (*n* = 102)	Obese (*n* = 81)	*P* value
**Demographic characteristics**				
Age at symptom onset, years	65.39 ± 8.96	66.60 ± 8.66	65.32 ± 9.24	0.516
Female,	76 (55.07%)	51 (50.00%)	41 (50.62%)	0.693
Years of education, years	9.22 ± 4.50	10.48 ± 4.88	8.67 ± 4.65	0.024^c^
Symptom duration, months	19.48 ± 17.70	15.88 ± 15.67	13.65 ± 13.04	0.027^b^
UPDRS-III	24.32 ± 10.66	22.05 ± 8.47	22.07 ± 8.65	0.111
MMSE	26.57 ± 2.62	26.64 ± 2.65	26.36 ± 2.84	0.778
Vascular risk factors				
Hypertension	54 (39.13%)	47 (46.08%)	40 (49.38%)	0.292
Diabetes mellitus	24 (17.39%)	20 (19.61%)	21 (25.93%)	0.310
TC levels	180.74 ± 38.72	184.52 ± 35.18	187.68 ± 37.06	0.398
Statin use	38 (27.54%)	31 (30.39%)	32 (39.51%)	0.176
WMHs burden	11.33 ± 8.75	9.89 ± 7.59	11.04 ± 7.31	0.374
Follow-up duration	5.29 ± 2.28	4.98 ± 2.38	5.77 ± 2.55	0.088
BMI	20.85 ± 1.49	23.91 ± 0.60	27.05 ± 1.71	< 0.001^a,b,c^
**Neuropsychological test**				
Attention/working memory	− 0.14 ± 0.87	− 0.13 ± 0.97	− 0.28 ± 0.81	0.458
Language	− 0.41 ± 1.12	− 0.24 ± 1.13	− 0.35 ± 1.16	0.525
Visuospatial	− 0.31 ± 1.47	− 0.70 ± 2.24	− 0.39 ± 1.57	0.215
Memory	− 0.36 ± 0.86	− 0.31 ± 0.89	− 0.29 ± 0.72	0.783
Frontal/executive	− 0.33 ± 0.93	− 0.25 ± 0.94	− 0.46 ± 0.75	0.296
CDR-SOB	1.16 ± 1.35	1.30 ± 1.61	1.22 ± 1.26	0.748
**Baseline DAT availability**				
Anterior caudate	2.06 ± 0.66	1.98 ± 0.68	2.15 ± 0.63	0.237
Posterior caudate	1.42 ± 0.58	1.30 ± 0.55	1.40 ± 0.52	0.250
Anterior putamen	2.21 ± 0.61	2.17 ± 0.60	2.29 ± 0.59	0.394
Posterior putamen	1.40 ± 0.44	1.38 ± 0.41	1.40 ± 0.45	0.936
Ventral putamen	1.46 ± 0.39	1.45 ± 0.40	1.50 ± 0.40	0.683
Ventral striatum	2.02 ± 0.56	2.04 ± 0.58	2.12 ± 0.57	0.446

Values are expressed as mean ± standard deviation or number (percentage)

*BMI* body mass index, *CDR-SOB* Clinical Dementia Rating-Sum of Boxes, *DAT* dopamine transporter, *MMSE* Mini-mental status examination, *TC* total cholesterol, *UPDRS-III* Unified Parkinson’s Disease Rating Scale Part III, *WMHs* white matter hyperintensities

^a^Significantly different in comparison between the under-/normal weight and overweight groups

^b^Significantly different in comparison between the overweight and obese groups

^c^Significantly different in comparison between the under-/normal weight and obese groups

### Relationship between TC levels, BMI, and cognitive function

There was no significant correlation between the MMSE score and TC levels (*R* = 0.08, *p* = 0.149) or BMI (*R* = -0.04, *p* = 0.490). Correlation analysis showed that TC levels were weakly associated with the composite score of frontal/executive function (*R* = 0.13, *p* = 0.022), whereas there was no significant relationship between BMI and the composite scores of attention, language, visuospatial, memory, and executive performance (Supplementary Figure S1). However, multivariable linear regression models showed that neither TC levels nor BMI were associated with the composite score of each cognitive domain after adjusting for covariates (Supplementary Table S1).

### Interaction effect of TC levels and BMI on cognition

**Fig. 1:**
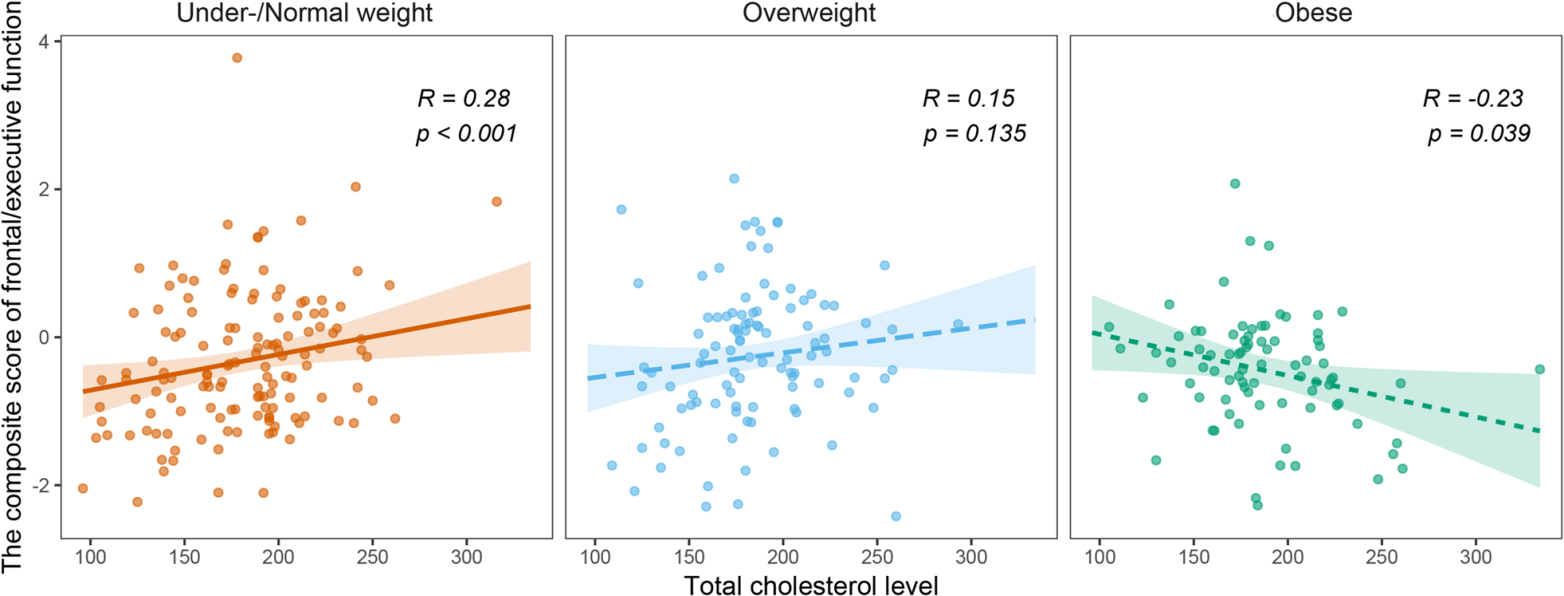
Scatter plots showing the composite score of frontal/executive function and TC levels according to BMI subgroups. A significant positive relationship is observed between TC levels and frontal/executive functions in the under-/normal weight group (red line), whereas TC levels are negatively associated with frontal/executive functions in the obese group (green line). BMI, body mass index; TC, total cholesterol

The results of the interaction analysis for each cognitive domain score are presented in Table 2. The interaction term between TC levels and BMI was significant for the frontal/executive function (*β* =  − 0.050, standard error [SE] = 0.018*, p* = 0.006, FDR-corrected *p* = 0.030) after adjusting for covariates (Table 2). Thus, we examined the relationship between TC levels and frontal/executive function according to the BMI groups. The subgroup analysis showed a significant positive relationship between TC levels and frontal/executive function in the under-/normal weight group (*R* = 0.28, *p* < 0.001), whereas TC levels were negatively associated with frontal/executive function in the obese group (*R* =  − 0.23, *p* = 0.039, Fig. 1). However, this association was not significant in the overweight group. In addition, we used regression analysis to examine the opposite relationship between TC levels and frontal/executive functions according to the BMI groups. After adjusting for confounding factors, TC levels were positively associated with frontal/executive function in under-/normal weight group (*β* = 0.205, SE = 0.082*, p* = 0.013) and negatively associated with frontal/executive function in the obese group (*β* =  − 0.213, SE = 0.087*, p* = 0.017, Table 3).

**Table 2:** Interaction analyses for the association of total cholesterol levels with each cognitive domain

Cognitive domain	Attention/working memory		Language		Visuospatial		Memory		Frontal/executive	
Variables	*β (SE)*	*P*	*β (SE)*	*P*	*β (SE)*	*P*	*β (SE)*	*P*	*β (SE)*	*P*
Intercept	-	-	-	-	-	-	-	-	0.413 (0.661)	0.533
Age	-	-	-	-	-	-	-	-	− 0.009 (0.006)	0.156
Female	-	-	-	-	-	-	-	-	0.141 (0.106)	0.185
Education	-	-	-	-	-	-	-	-	0.010 (0.011)	0.343
Symptom duration	-	-	-	-	-	-	-	-	− 0.001 (0.003)	0.764
Hypertension	-	-	-	-	-	-	-	-	0.085 (0.109)	0.435
Diabetes	-	-	-	-	-	-	-	-	− 0.224 (0.127)	0.079
Statin use	-	-	-	-	-	-	-	-	0.022 (0.111)	0.841
DAT-PP										
WMHs burden	-	-	-	-	-	-	-	-	− 0.019 (0.007)	0.006
BMI	-	-	-	-	-	-	-	-	− 0.011 (0.018)	0.523
TC levels per 1 SD increase	-	-	-	-	-	-	-	-	1.220 (0.426)	0.004
BMI × TC levels	− 0.020 (0.018)	0.275	− 0.035 (0.022)	0.115	− 0.022 (0.038)	0.557	− 0.017 (0.017)	0.325	− **0.050 (0.018)**	**0.006**

Interaction analyses were performed to investigate the interaction effect between total cholesterol levels and other dependent variables (age at symptom onset, sex, years of education, symptom duration, hypertension, diabetes, statin use, white matter hyperintensities, and BMI) on each cognitive domain. Each interaction analysis was performed after adjusting for age at symptom onset, sex, years of education, symptom duration, the presence of hypertension and diabetes, statin use, white matter hyperintensities, and BMI

*β* regression coefficient, *BMI* body mass index, *DAT-PP* dopamine transporter availability in the posterior putamen, *SD* standard deviation, *SE* standard error, *TC* total cholesterol, *WMHs* white matter hyperintensities

Bold indicates FDR-corrected *P* < 0.05

**Table 3:** Multivariate linear regression analyses for the association of total cholesterol levels with frontal/executive function according to body mass index

Variables	Under-/normal weight (BMI < 23)		Overweight (23 ≤ BMI < 25)		Obese (BMI > 25)	
	*β (SE)*	*P*	*β (SE)*	*P*	*β (SE)*	*P*
Intercept	− 1.088 (1.657)	0.513	5.591 (3.512)	0.115	0.407 (1.599)	0.800
Age	0.002 (0.011)	0.822	− 0.024 (0.011)	0.036	− 0.009 (0.011)	0.429
Female	0.104 (0.174)	0.550	0.266 (0.199)	0.184	− 0.008 (0.191)	0.967
Education	0.012 (0.019)	0.528	0.019 (0.019)	0.333	− 0.004 (0.021)	0.835
Symptom duration	0.001 (0.004)	0.802	− 0.003 (0.006)	0.649	− 0.001 (0.007)	0.930
Hypertension	0.047 (0.188)	0.802	− 0.061 (0.199)	0.761	0.356 (0.2)	0.080
Diabetes	− 0.37 (0.231)	0.112	− 0.159 (0.228)	0.488	− 0.19 (0.199)	0.343
Statin use	0.392 (0.195)	0.047	− 0.183 (0.208)	0.381	− 0.325 (0.186)	0.084
DAT-PP						
WMHs burden	− 0.021 (0.01)	0.045	− 0.022 (0.013)	0.095	− 0.025 (0.014)	0.084
BMI	0.024 (0.058)	0.683	− 0.186 (0.143)	0.198	0.003 (0.052)	0.953
TC levels per 1 SD increase	**0.205 (0.082)**	**0.013**	0.078 (0.099)	0.436	− **0.213 (0.087)**	**0.017**

Multivariate linear regression models were used to investigate the association between total cholesterol levels and cognition, while adjusting for age at symptom onset, sex, years of education, symptom duration, the presence of hypertension and diabetes, statin use, white matter hyperintensities, and BMI

*β* regression coefficient, *BMI* body mass index, *DAT-PP* dopamine transporter availability in the posterior putamen, *SD* standard deviation, *SE* standard error, *TC* total cholesterol, *WMHs* white matter hyperintensities

### Relationship between TC levels, BMI, and PDD conversion

**Fig. 2:**
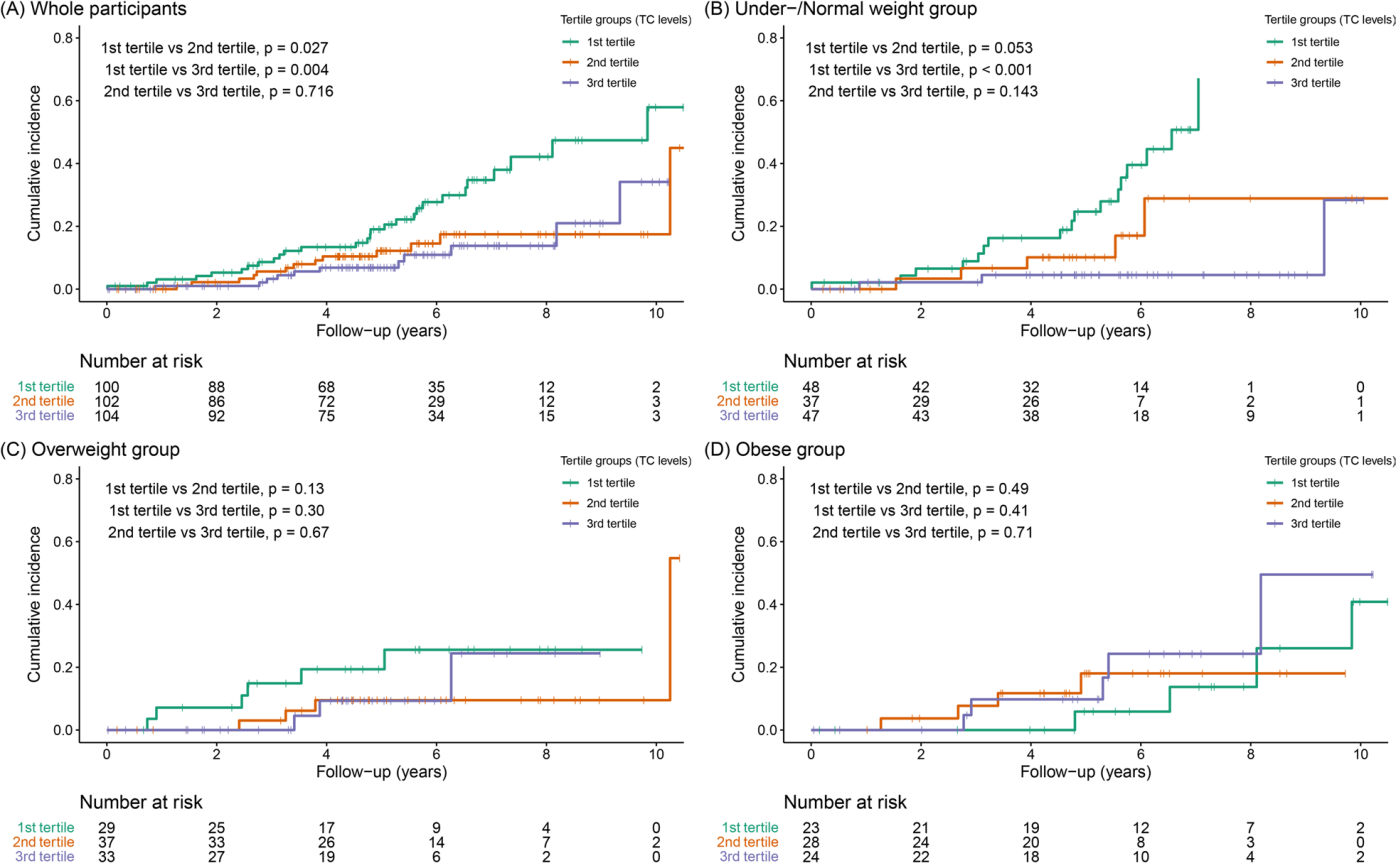
Kaplan–Meier survival curve of PDD conversion. The crosses in the graphs indicate censored data

Among the 306 patients with PD, 26 (19.7%), 13 (13.5%), and 13 (16.7%) patients in the under-/normal weight, overweight, and obese groups, respectively, developed PDD during the follow-up period. The follow-up duration was 5.3 ± 2.3, 5.0 ± 2.4, and 5.8 ± 2.6 in the under-/normal weight, overweight, and obese groups, respectively. Kaplan–Meier analysis revealed that the 1st tertile group of TC levels had a higher risk of PDD conversion than that of the 2nd and 3rd tertile groups (*P*_*log-rank*_ = 0.027 and 0.004, respectively; Fig. 2A). However, the Cox regression model showed that neither TC levels nor BMI predicted PDD conversion after adjusting for confounding factors (Table 4, *Model 1*). Interaction analysis showed that the interaction term between TC levels and BMI was significant for PDD conversion (Table 4, *Model 2*). Thus, we further examined the relationship between TC levels and PDD conversion according to the BMI group. In subgroup analysis according to the BMI groups, Kaplan–Meier analyses showed that 1st tertile group of TC levels had a higher risk of PDD conversion than that of 3rd tertile groups (*P*_*log-rank*_ < 0.001, Fig. 2B) in the under-/normal weight subgroup, whereas the risk of PDD conversion did not differ between the tertile groups of TC levels in the overweight and obese subgroups (Fig. 2C and D). In the Cox regression models, higher TC levels were associated with a lower future risk of PDD conversion in the under-/normal weight group (HR = 0.550, 95% CI = 0.344–0.880, *p* = 0.013) and a higher future risk of PDD conversion in the obese group (HR = 2.085, 95% CI = 1.160–3.746, *p* = 0.014) after adjusting for covariates (Table 5).

**Table 4:** Cox regression analysis of the effects of total cholesterol levels on dementia conversion

Variables	**Model 1 (C-index = 0.791)**		**Model 2 (C-index = 0.793)**	
	HR (95% CI)	*P*	HR (95% CI)	*P*
Age at symptom onset	1.063 (1.023–1.105)	0.002	1.068 (1.027–1.111)	0.001
Female	0.381 (0.188–0.771)	0.007	0.367 (0.183–0.736)	0.005
Symptom duration	1.003 (0.984–1.023)	0.736	1.001 (0.981–1.02)	0.958
Education	0.999 (0.933–1.070)	0.973	0.997 (0.931–1.066)	0.919
MMSE score	0.808 (0.710–0.919)	0.001	0.795 (0.695–0.909)	0.001
Hypertension	1.333 (0.725–2.449)	0.355	1.439 (0.782–2.649)	0.242
Diabetes	0.864 (0.442–1.687)	0.668	0.774 (0.392–1.527)	0.459
Statin use	1.513 (0.799–2.866)	0.204	1.489 (0.790–2.807)	0.218
DAT-PP				
WMHs burden	1.025 (0.991–1.061)	0.150	1.025 (0.991–1.061)	0.150
BMI	0.930 (0.837–1.033)	0.177	0.977 (0.872–1.096)	0.696
TC levels per 1 SD increase	0.863 (0.638–1.166)	0.336	0.015 (0.001–0.154)	< 0.001
BMI × TC levels	**-**	**-**	**1.193 (1.081**–**1.317)**	** < 0.001**

Results of Cox regression analyses for PDD conversion after controlling for age at symptom onset, sex, symptom duration, years of education, MMSE scores, the presence of hypertension and diabetes, statin use, WMHs burden, BMI, TC levels, and/or the interaction term of BMI*TC levels

*BMI* body mass index, *CI* confidence interval, *DAT-PP* dopamine transporter availability in the posterior putamen, *HR* hazard ratio, *MMSE* the Mini-Mental state Examination, *TC* total cholesterol, *WMHs* white matter hyperintensities

**Table 5:** Cox regression analysis of the effects of total cholesterol levels on dementia conversion according to body mass index

Variables	Under-/normal weight (C-index = 0.816)		Overweight (C-index = 0.884)		Obese (C-index = 0.86)	
	HR (95% CI)	*P*	HR (95% CI)	*P*	HR (95% CI)	*P*
Age at symptom onset	1.044 (0.981–1.110)	0.174	1.122 (1.003–1.256)	0.045	1.155 (1.009–1.322)	0.037
Female	0.206 (0.067–0.630)	0.006	0.263 (0.032–2.15)	0.213	0.171 (0.032–0.916)	0.039
Symptom duration	1.003 (0.975–1.031)	0.85	1.004 (0.969–1.041)	0.813	0.995 (0.933–1.062)	0.882
Education	1.011 (0.902–1.132)	0.856	0.970 (0.816–1.153)	0.727	0.846 (0.689–1.037)	0.107
MMSE score	0.633 (0.504–0.794)	< 0.001	0.907 (0.643–1.279)	0.577	0.912 (0.703–1.184)	0.490
Hypertension	0.718 (0.247–2.089)	0.543	9.886 (1.471–66.428)	0.018	2.497 (0.439–14.211)	0.302
Diabetes	1.229 (0.397–3.805)	0.720	0.076 (0.008–0.774)	0.030	1.370 (0.321–5.850)	0.671
Statin use	1.015 (0.393–2.619)	0.975	2.833 (0.575–13.961)	0.201	0.798 (0.204–3.123)	0.746
DAT-PP						
WMHs burden	1.000 (0.955–1.048)	0.993	1.036 (0.963–1.115)	0.338	1.124 (1.019–1.241)	0.019
BMI	0.867 (0.612–1.230)	0.424	0.981 (0.303–3.181)	0.975	0.947 (0.556–1.613)	0.841
TC levels per 1 SD increase	**0.550 (0.344**–**0.880)**	**0.013**	0.908 (0.399–2.065)	0.818	**2.085 (1.160**–**3.746)**	**0.014**

Results of Cox regression analyses for PDD conversion after controlling for age at symptom onset, sex, symptom duration, years of education, MMSE scores, the presence of hypertension and diabetes, statin use, WMHs burden, BMI, and TC levels

*BMI* body mass index, *CI* confidence interval, *DAT-PP* dopamine transporter availability in the posterior putamen, *HR* hazard ratio, *MMSE* the Mini-Mental state Examination, *TC* total cholesterol, *WMHs* white matter hyperintensities

## Discussion

This study investigated the complex relationship among TC levels, BMI, and cognition in drug-naïve patients with PD. The major findings were as follows: (i) baseline cognitive function and longitudinal cognitive outcomes were not associated with baseline TC level and BMI; however, there was a significant interaction effect between TC levels and BMI for frontal/executive function and PDD conversion; (ii) lower TC levels were associated with lower composite scores of frontal/executive function and a higher future risk of PDD conversion in the under-/normal weight group, whereas higher TC levels were associated with poor performance on frontal/executive items and more frequent PDD conversion in the obese group. These findings indicate that the association between TC levels and cognition is moderated by BMI in patients with PD, suggesting that a cholesterol-lowering strategy could be applied differentially according to BMI.

Clinical studies have demonstrated that individuals with high levels of circulating TC are less likely to develop PD and have better long-term outcomes. [10, 23, 24]. However, high cholesterol levels can worsen the loss of dopaminergic neurons in a PD model [25]. This is because cholesterol facilitates the interaction between oligomeric α-synuclein and the cell membrane, leading to membrane disruption and cell death. A recent investigation discovered that elevated cholesterol levels have a dual role in PD: they protect against lysosomal membrane permeabilization while also promoting α-synuclein accumulation [26]. In terms of BMI, although results from several epidemiologic studies are conflicting about the association between BMI and PD risk [27, 28], low BMI or weight loss is associated with dopaminergic neuronal degeneration or cognitive outcomes in patients with PD [12–14]. To date, no study has investigated how BMI and TC levels interact with each other and their effect on cognition in patients with PD.

Although the present study showed a similar relationship between baseline TC levels and frontal/executive function in a simple correlation analysis, this relationship was no longer significant after adjusting for covariates. However, we found a significant interaction between TC levels and BMI for frontal/executive functions, indicating that TC levels differentially affect frontal/executive functions according to BMI. Consistent with the findings from a previous study, further subgroup analyses revealed that higher TC levels were associated with higher composite scores of frontal/executive function in the under-/normal weight group [10]. In contrast, TC levels were negatively associated with frontal/executive function in the obese group. This result suggests that, although the mechanism remains speculative, TC levels can negatively contribute to cognitive performance in obese patients with PD, similar to the inverse association between TC levels and cognition in young adults reported in previous studies [29, 30].

The longitudinal analysis of PDD conversion showed results similar to those of the cross-sectional analysis. Although TC levels or BMI could not predict PDD conversion in the Cox regression model after adjusting for confounding factors, a significant interaction effect was observed between TC levels and BMI for PDD conversion. In the subgroup analyses, higher TC levels were associated with a lower future risk of PDD conversion in the under-/normal weight group, while higher TC levels were associated with more frequent PDD conversion in the obese group. When examining the association between cholesterol levels and Alzheimer’s disease or vascular dementia, previous studies have shown inconsistent results, and this association seems to vary with age at measurement (mid-life [< 65 years] or later life [≥ 65 years]) and follow-up duration [31, 32]. In this study, we investigated whether there was a significant interaction between TC levels and each demographic variable, including age, sex, hypertension, diabetes, statin use, and BMI (data not shown), in which only the interaction effect between TC levels and BMI was significant. This is the first study to show that TC levels in patients with PD are associated with baseline cognition and the future risk of dementia conversion in a BMI-dependent manner. Causal relationships could not be assessed in this study, and reverse causation should also be considered. For example, because TC levels are biomarkers of malnutrition [33], patients with PD who have low TC levels in the under-/normal weight group may be malnourished, which subsequently leads to poor cognitive outcome. In addition, a recent study showed that familiar patients with PD, particularly those with a glucocerebrosidase (GBA) mutation, have lower cholesterol levels than healthy controls and patients with sporadic PD [34]. Considering the fact that GBA is associated with poor cognitive performance and the risk of PDD [35], poor cognitive outcomes in patients with PD and low TC levels in the under-/normal weight group may be attributed to underlying genetic risk factors [36]. Meanwhile, the relationship between metabolic syndrome and dementia could provide a possible explanation for the opposite association between TC levels and cognitive outcomes in the obese group. Although TC levels are not included in the diagnostic criteria for metabolic syndrome, they are elevated in patients with metabolic syndrome [37]. Because metabolic syndrome is associated with the risk of AD [38] and TC levels exert an additive effect on the association between obesity and dementia risk [39], high TC levels in the obese PD group may have detrimental effects on cognitive outcome through AD or vascular co-pathology.

Our study had some limitations. First, 24S-hydroxycholesterol and 27S-hydroxycholesterol, which are indicators of brain cholesterol levels, were not analyzed. While this study analyzed TC levels, which correlated well with brain cholesterol content [40], future research using brain cholesterol biomarkers is required to confirm these findings. Second, the study did not have access to data on LDL, high-density lipoprotein cholesterol, and triglyceride levels, which are components of TC; therefore, further research involving larger cohorts is required to determine which fraction of cholesterol affects cognitive symptoms in patients with PD. Third, although the study adjusted for statin use, some degree of under-adjustment may have occurred because of the absence of information on the duration and dosage of statin exposure in this retrospective study. Finally, because this was a single-center study in Korea, the generalization of results is limited by factors such as ethnicity and genetic background.

## Conclusions

In conclusion, our study demonstrated that the relationship between TC levels and cognition in patients with PD was influenced by BMI and differed between subgroups. Lower lipid levels in the under-/normal weight group and higher lipid levels in the obese group were associated with poor performance on frontal/executive items and the higher risk of PDD conversion. These findings suggest that cholesterol-lowering strategy could be applied differentially according to BMI in patients with PD.

### Supplementary Information

**Additional file 1: Supplementary Methods. Supplementary Figure S1.** Scatter plots showing the composite score of each cognitive domain and TC levels or BMI. **Supplementary Table S1.** Multivariate linear regression analyses for the association of total cholesterol levels with each cognitive domain.

## Data Availability

Data generated or analyzed during the study are available from the corresponding author by request.

## Abbreviations

AD: Alzheimer’s disease
ADL: Activities of daily living
BMI: Body mass index
CDR-SOB: Clinical Dementia Rating-Sum of Boxes
DAT: Dopamine transporter
DAT-PP: Dopamine transporter availability in the posterior putamen
FDR: False discovery rate
FLAIR: Fluid-attenuated inversion recovery
^18^F-FP-CIT: [^18^F] N-(3-fluoropropyl)-2β-carbonethoxy-3β-(4-iodophenyl) nortropane
GBA: Glucocerebrosidase
HRs: Hazard ratios
LDL: Low-density lipoprotein
MMSE: Mini-mental state examination
MRI: Magnetic resonance imaging
PD: Parkinson’s disease
PDD: Parkinson’s disease dementia
PET: Positron emission tomography
SD: Standard deviation
SE: Standard error
TC: Total cholesterol
UPDRS-III: Unified Parkinson’s Disease Rating Scale Part III
WMHs: White matter hyperintensities
