# Supplementary material for: Differential effects of cholesterol levels on cognition according to body mass index in Parkinson’s disease
Source: Alzheimers Res Ther. 2024 Jan 31;16:24. doi: 10.1186/s13195-023-01326-2 (PMC10829366; doi:10.1186/s13195-023-01326-2)
Supplement: Supplementary file 1 — Additional file 1: Supplementary Methods. Supplementary Figure S1. Scatter plots showing the composite score of each cognitive domain and TC levels or BMI. Supplementary Table S1. Multivariate linear regression analyses for the association of total cholesterol levels with each cognitive domain. [file 13195_2023_1326_MOESM1_ESM.docx]

**Supplementary Method S1. Acquisition and quantitative analyses of ^18^F-FP-CIT PET**

The ^18^F-FP-CIT PET scans were acquired using a GE PET-CT DSTe scanner (GE Discovery STE; GE Healthcare; Milwaukee, WI, USA), which obtains images with a three-dimensional resolution of 2.3-mm full width at half maximum. After the subjects fasted for at least 6 h, they were intravenously injected with 5mCi (185 MBq) of ^18^F-FP-CIT. 90 min after the injection, PET images were acquired for 20 min in the three-dimensional mode at 12 kVp and 380 mA. Image processing was performed using SPM8 (Wellcome Department of Imaging Neuroscience, Institute of Neurology, UCL, London, UK) with Matlab 2013a for Windows (Math Works, Natick, MA, USA). Quantitative analyses were based on volumes of interests (VOIs), which were defined based on a template in standard space. All reconstructed PET images were spatially normalized to the Montreal Neurology Institute (MNI) template space using a standard ^18^F-FP-CIT PET template which was generated from ^18^F-FP-CIT PET and T1-weighted MRI scans of 13 normal controls. Twelve VOIs of bilateral striatal subregions and one occipital VOI were drawn on a co-registered spatially normalized single T1-weighted MR and ^18^F-FP-CIT PET template image on MRIcro version 1.37 (Chris Rorden, Columbia, SC, USA).^1^ Briefly, the striatum was divided along the anterior-posterior commissure line on the transverse plane into dorsal and ventral portions. The ventral portion was comprised of two subregions: the ventral putamen and ventral striatum. Subsequently, the dorsal portion was divided along the coronal anterior commissure plane into the following anterior and posterior subregions: the anterior caudate, posterior caudate, anterior putamen, and posterior putamen.^2^ These VOIs were adjusted by a minor translation in our in-house editing software ANIQUE. DAT availability was calculated by the non-displaceable binding potential, which was defined as follows: (mean standardized uptake value of the striatal subregions VOI–mean standardized uptake value of the occipital VOI)/(mean standardized uptake of the occipital VOI).^3^

**Reference**

1. Jeong SH, Lee HS, Jung JH, et al. White Matter Hyperintensities, Dopamine Loss, and Motor Deficits in De Novo Parkinson's Disease. Mov Disord 2021;36(6):1411-1419.

2. Oh JS, Oh M, Chung SJ, Kim JS. Cerebellum-specific 18F-FDG PET analysis for the detection of subregional glucose metabolism changes in spinocerebellar ataxia. Neuroreport 2014;25(15):1198-1202.

3. Jeong SH, Chung SJ, Yoo HS, et al. Beneficial effects of dipeptidyl peptidase-4 inhibitors in diabetic Parkinson's disease. Brain 2021;144(4):1127-1137.

**Supplementary Figure S1. Scatter plots showing the composite score of each cognitive domain and TC levels or BMI**

**
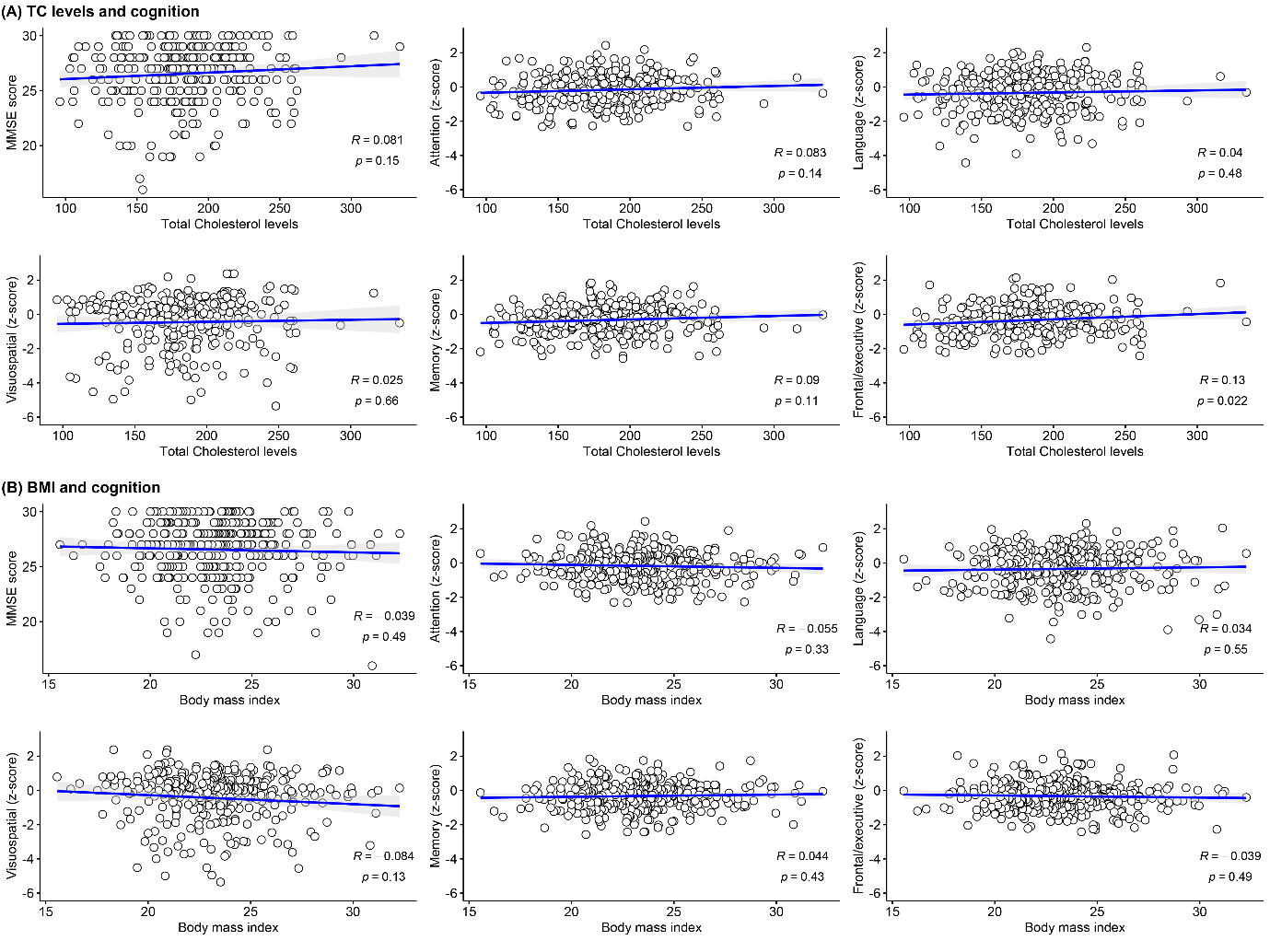
**

Blue line = linear trend; light blue band = 95% confidence interval

BMI = body mass index; TC = total cholesterol

**Supplementary Table S1. Multivariate linear regression analyses for the association of total cholesterol levels with each cognitive domain**

| Cognitive domain | Attention/Working Memory | | Language | | Visuospatial | | Memory | | Frontal/executive | |
| --- | --- | --- | --- | --- | --- | --- | --- | --- | --- | --- |
| Variables | *β (SE)* | *P* | *β (SE)* | *P* | *β (SE)* | *P* | *β (SE)* | *P* | *β (SE)* | *P* |
| Intercept | 1.324 (0.687) | 0.055 | 1.784 (0.867) | 0.040 | -0.302 (1.461) | 0.837 | -0.524 (0.619) | 0.398 | 0.203 (0.709) | 0.775 |
| Age | -0.021 (0.006) | 0.001 | -0.023 (0.008) | 0.003 | 0.002 (0.013) | 0.868 | -0.008 (0.006) | 0.152 | -0.009 (0.006) | 0.148 |
| Female | 0.160 (0.103) | 0.122 | -0.398 (0.103) | 0.002 | 0.215 (0.219) | 0.327 | 0.274 (0.099) | 0.006 | 0.168 (0.106) | 0.116 |
| Education | 0.026 (0.011) | 0.017 | 0.002 (0.014) | 0.898 | 0.037 (0.023) | 0.104 | 0.023 (0.010) | 0.023 | 0.011 (0.011) | 0.328 |
| Symptom duration | -0.002 (0.003) | 0.447 | -0.001 (0.004) | 0.868 | 0.002 (0.006) | 0.746 | -0.001 (0.003) | 0.822 | -0.001 (0.003) | 0.699 |
| Hypertension | -0.027 (0.107) | 0.803 | -0.066 (0.134) | 0.623 | -0.289 (0.227) | 0.202 | -0.011 (0.102) | 0.915 | 0.101 (0.110) | 0.359 |
| Diabetes | -0.236 (0.124) | 0.059 | -0.223 (0.157) | 0.156 | -0.39 (0.264) | 0.141 | -0.059 (0.119) | 0.621 | -0.247 (0.128) | 0.055 |
| Statin use | 0.072 (0.109) | 0.507 | 0.446 (0.137) | 0.001 | 0.169 (0.231) | 0.465 | 0.083 (0.104) | 0.425 | 0.037 (0.112) | 0.745 |
| DAT-PP |  |  |  |  |  |  |  |  |  |  |
| WMHs burden | -0.015 (0.007) | 0.029 | -0.030 (0.008) | <0.001 | 0.000 (0.014) | >0.999 | -0.020 (0.006) | 0.002 | -0.02 (0.007) | 0.003 |
| BMI | -0.016 (0.017) | 0.352 | 0.003 (0.022) | 0.873 | -0.036 (0.037) | 0.325 | 0.014 (0.016) | 0.403 | -0.014 (0.018) | 0.429 |
| TC levels per  1 SD increase | -0.001 (0.049) | 0.978 | 0.030 (0.062) | 0.636 | 0.003 (0.105) | 0.979 | 0.013 (0.047) | 0.790 | 0.053 (0.051) | 0.303 |

Multivariate linear regression models were used to investigate the association between total cholesterol levels and cognition, while adjusting for age at symptom onset, sex, years of education, symptom duration, the presence of hypertension and diabetes, statin use, white matter hyperintensities, and BMI.

*β* = regression coefficient; SE = standard error.
